# The Immunogenicity of a VLP-based Malaria Vaccine Targeting CSP in Pregnant and Neonatal Mice

**DOI:** 10.3390/biom13020202

**Published:** 2023-01-19

**Authors:** Lucie Jelínková, Bryce Roberts, Diane T. Ajayi, David S. Peabody, Bryce Chackerian

**Affiliations:** Department of Molecular Genetics and Microbiology, University of New Mexico School of Medicine, Albuquerque, NM 87131, USA

**Keywords:** maternal antibodies, neonatal vaccination, animal models, malaria vaccines, circumsporozoite protein

## Abstract

Maternal antibodies are passively transferred to the fetus via the placenta during gestation and can play an important role in protecting the newborn from infection. For example, in malaria-endemic regions, maternal antibodies likely provide substantial protection against *Plasmodium falciparum* malaria in the first 6 months of life. However, circulating maternal antibodies can also interfere with vaccine efficacy. Here, we used a mouse maternal transfer model to evaluate whether maternal antibodies interfere with the responsiveness to a virus-like particle (VLP)-based vaccine targeting the CIS43 epitope of the malaria circumsporozoite protein (CSP). We found immunized dams passively transfer to pups high levels of anti-CSP IgG antibodies that steadily decline as the animals age. We also found that the neonatal offspring of immunized mice do not respond to de novo immunization with the CIS43-targeted VLP vaccine until maternal antibody titers decline below an inhibitory threshold. These findings may have important implications for delineating the delicate balance between protection conferred by maternal antibodies and the offspring’s ability to respond to immunization.

## 1. Introduction

Neonates are born with an immature immune system, increasing their vulnerability to infections [1]. However, the transfer of maternal antibodies through the placenta to fetuses during the third trimester of pregnancy can confer protection against infection for roughly the first six months of life [2]. Studies in animal models and humans have shown that transferred maternal antibodies can provide protection against diverse human pathogens, including influenza [3,4,5], herpes simplex virus [6], *Bordetella pertussis* [7], and many others. Thus, vaccination of pregnant women can be an effective strategy for decreasing neonatal susceptibility to infection. For example, a Tdap vaccine booster is routinely administered to pregnant women to ensure that infants have protective levels of antibodies against diphtheria, tetanus, and pertussis until they are old enough to receive the Tdap vaccine [8]. Similarly, maternal vaccination has been proposed as an approach to provide protection against other pathogens to which neonates and infants are particularly vulnerable, such as respiratory syncytial virus [9]. However, as maternal antibody levels decay, their ability to mediate protection wanes. 

In addition to their protective role, high concentrations of maternal antibodies can also interfere with the response of infants to vaccination [10,11,12,13], adding an additional layer of complexity to the immunoprophylaxis of diseases that disproportionately affect the young. The negative effects of maternal antibodies have been observed with several vaccines, including protein-based vaccines for tetanus and pneumococcus [14], the live-attenuated mumps virus vaccine [15] and others [11,13]. Perhaps the best-studied example is the measles vaccine, which is less effective when used in young children with pre-existing maternal antibodies [16]. Efforts to circumvent maternal antibody-mediated inhibition of the measles vaccine have included the use of increased doses of vaccine [17] and modifications to the vaccine administration schedule [15].

*Plasmodium falciparum (Pf)* malaria is a major cause of worldwide morbidity and mortality, particularly in young children under 5 years of age in sub-Saharan Africa [18]. However, neonates and infants under 1 year of age born in malaria-endemic regions of the world are at lower risk of severe disease [19,20,21,22] and exhibit lower blood parasitemia as well as higher rates of clearance compared to older children [23,24]. This phenomenon is hypothesized to be due to maternal anti-*Pf* antibodies in mothers repeatedly exposed to malaria [21,25,26]. However, the protective value of maternal antibodies in malaria is still controversial; some studies suggested that anti-malaria antibodies in neonates and infants are a simply a biomarker of exposure rather than evidence of protective maternal antibody transfer [21,25]. 

We developed vaccines targeting the epitopes of two anti-*Pf* circumsporozoite protein (CSP) monoclonal antibodies (mAbs), called CIS43 and L9 [27,28], which are amongst the most potent mAbs at providing protection from experimental malaria challenge in mouse models [29,30]. Passive immunization with CIS43 and L9 provides complete protection from controlled human malaria infection [31,32] and a recent Phase 2 clinical trial in Mali demonstrated that a single dose of CIS43 was strongly protective against *Pf* infection over a 6-month malaria season [33]. Our vaccines display the CIS43 and L9 epitopes multivalently on the surface of bacteriophage VLPs and elicit durable high-titer anti-CSP antibodies that protect mice from malaria challenge [27,28]. The experiments described below were designed to determine whether maternal antibodies of vaccinated mice interfere with de novo immunization of their pups and whether such interference could be overcome by immunization with higher doses, by presenting the antigen on a distinct vaccine platform, or by delaying immunization. These studies provide guidelines that may govern the use of VLP-based immunogens in neonates.

## 2. Materials and Methods

*VLP production:* Qβ and MS2(S99K) bacteriophage VLPs were expressed by transforming C41 *E. coli* cells with pET-derived plasmids encoding the Qß and MS2 bacteriophage viral coat proteins. MS2(S99K) is a modified version of the MS2 coat protein in which the number of surface-exposed lysines on the VLPs has been increased by changing the surface-exposed serine residue at amino acid 99 to a lysine. Transformed C41 cells were grown at 37 °C using Luria Bertani broth containing 60 µg/mL kanamycin until the cells reached an OD600 of 0.6. Coat protein expression was induced using 0.4 mM isopropyl-β-D-1-thiogalactopyranoside (IPTG) and grown at 37 °C for 3 h. Cell pellets were collected and re-suspended using a lysis buffer (50 mM Tris-HCL, 100 mM NaCl, 10 mM EDTA, pH 8.5). Cells were lysed by sonication and cell lysates were clarified by centrifugation (15,000× *g*, 20 min, 4 °C). Soluble recombinant MS2 VLPs were purified by precipitation using 70% saturated (NH_4_)_2_SO_4_, followed by size exclusion chromatography (SEC) using a Sepharose CL-4B column. The column was pre-equilibrated with a purification buffer (40 mM Tris-HCl, 400 mM NaCl, 8.2 mM MgSO_4_, pH 7.4). VLPs were concentrated from SEC purified fractions by precipitation using 70% saturated (NH_4_)_2_SO_4_ and then extensively dialyzed versus PBS, pH 7.4. VLPs were depleted of endotoxin by multiple rounds of phase separation using Triton X-114 (Sigma-Aldrich, St. Louis, MO, USA), as described in [34].

A synthetic CIS43 peptide containing a four-amino acid C-terminal linker sequence (NPDPNANPNVDPNAN*GGGC*) was displayed on VLPs as described in [28]. Briefly, the peptide was conjugated directly to surface lysines on VLPs using the bidirectional crosslinker succinimidyl 6-[(β-maleimidopropionamido) hexanoate] (SMPH; Thermo Fisher Scientific, Waltham MA, USA).

*Mouse immunization and maternal transfer studies:* All animal research was approved by the Institutional Animal Care and Use Committee of the University of New Mexico School of Medicine (protocol number: 19-200870-HSC). In most experiments described in the manuscript, Balb/c mice were immunized intramuscularly with 5 µg of CIS43 VLPs without exogenous adjuvant; in some experiments, a higher dose (20 µg) was used. Sera were collected at multiple timepoints post-immunization by tail vein or by retroorbital bleed, depending on the age of the mice. At some timepoints, sera were collected and mice were immunized; on these occasions, sera were collected prior to immunization.

For maternal transfer studies, adult female Balb/c mice were immunized intramuscularly with 5 µg CIS43 VLPs without exogenous adjuvant three times at three-week intervals. Following the immunization series, mice were allowed to breed with a single male Balb/c mouse. Once pregnancy was confirmed in the female mice, the male was separated from the cage. Litters were allowed to nurse for 2–3 weeks, then blood samples were collected by tail vein bleeds and sera was evaluated for anti-CSP IgG by ELISA. Progeny males and females were separated post-weaning at about 3 weeks of age, and anti-CSP IgG was re-evaluated by ELISA at multiple timepoints post-weaning using sera collected by retroorbital bleeds. Negative control sera were obtained from unimmunized (naïve) mice.

*Antibody Quantification:* Anti-CSP IgG titers were determined by ELISA using methods previously described [28]. Immulon 2 plates (Thermo Fisher Scientific, Waltham MA, USA) were coated with 250 ng of recombinant CSP expressed in *Pseudomonas fluorescens* [35] in 50 μL phosphate-buffered saline (PBS) and incubated at 4 °C overnight. Following incubation, wells were blocked with PBS-0.5% milk for 2 h at room temperature. Serum isolated from each individual mouse (including immunized mice and naïve controls) was serially diluted in PBS-0.5% milk and applied to wells and incubated at room temperature for 2.5 h. Reactivity to the target antigen was detected using HRP-labeled goat anti-mouse IgG (Jackson ImmunoResearch, West Grove PA, USA; diluted 1:4000). The reaction was developed using 3,3′,5,5′-tetramethylbenzidine (TMB) substrate (Thermo Fisher Scientific) and stopped using 1% HCl. Plates were read at 450 nm (OD_450_) using an AccuSkan plate reader (Fisher Scientific, Hampton NH, USA). End-point dilution titer was defined as the greatest sera dilution that yielded an OD_450_ > 2-fold over background. 

*Statistical Analysis:* End-point dilution titers were compared statistically by log-transforming values and then performing two-tailed t-tests using Prism version 9 (GraphPad Software, San Diego, CA, USA). 

## 3. Results

### 3.1. CIS43 VLP Immunization of Female Mice Elicits Anti-CSP IgG That is Transferred to Their Pups

**Figure 1:**
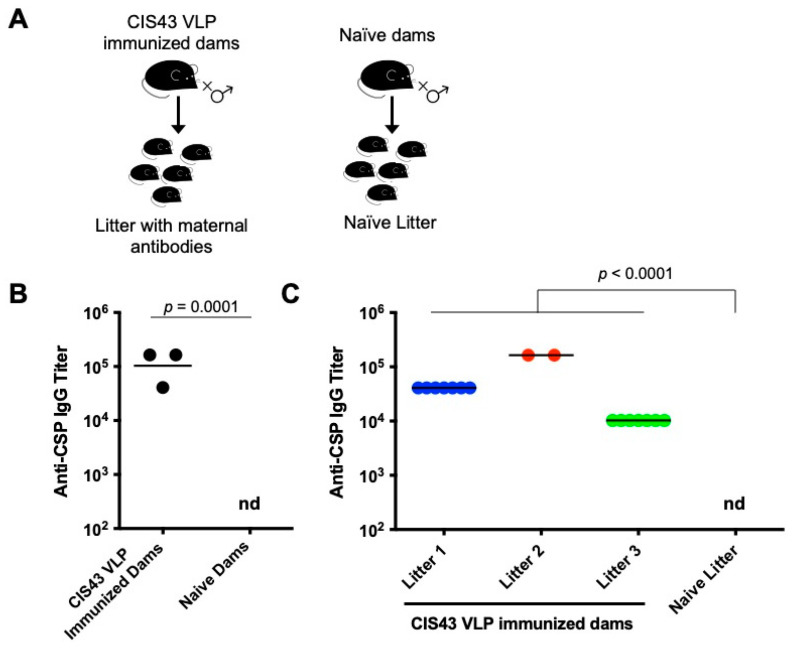
Anti-CSP IgG levels in CIS43 VLP-immunized dams and their pups. (**A**) A schematic of the experimental setup. Female Balb/c mice (*n* = 3) were immunized with three doses of 5 µg of CIS43 VLPs and then mated with a male mouse. A control group of unimmunized mice were also mated with a male mouse. (**B**) IgG titers against CSP in immunized mice were determined by ELISA two weeks following the third dose. A group of three control mice were used as negative controls. (**C**) Immediately following their final immunization, female mice were bred with a naïve Balb/c male. Each of the three dams gave birth to a litter with 7, 2, and 7 pups, respectively. Blood samples were collected from pups at about 2 weeks of age and anti-CSP IgG titers were determined by ELISA. As a negative control, three pups from the litter of a control dam were also tested. Each data point represents an individual mouse and lines denote the geometric mean titer of each group. Colors represent the different litters. If antibody titers were <40, the limit of detection, then the titer is listed as nd (not detected). Data were compared statistically by two-tailed *t* test using log-transformed values.

To assess the potential role of maternal antibodies in the context of an epitope-specific pre-erythrocytic malaria vaccine, we established a mouse maternal transfer model (Figure 1A). Female mice were immunized with a Qß VLP-based vaccine targeting the CIS43 epitope. This epitope is located at the junctional region between the N-terminal and central repeat regions of CSP and is the target of a potently protective anti-CSP monoclonal antibody [29,31]. Vaccination with CIS43 VLPs elicited high titer anti-CSP IgG responses (Figure 1B), similar to what we have previously reported [28]. Each vaccinated female mouse was then co-housed with a male mouse. Following breeding, progeny were housed with the nursing dams until they were weaned at three weeks of age. Two weeks after birth, sera were collected from pups and tested for anti-CSP IgG. Offspring mice exhibited high anti-CSP antibody titers (Figure 1C). While anti-CSP antibody levels were different between litters, likely reflecting differences in antibody levels in their mothers, they were remarkably consistent between individual pups in each litter.

### 3.2. Young Mice with Anti-CSP Maternal Antibodies Fail to Respond to Immunization with CIS43 VLPs

**Figure 2:**
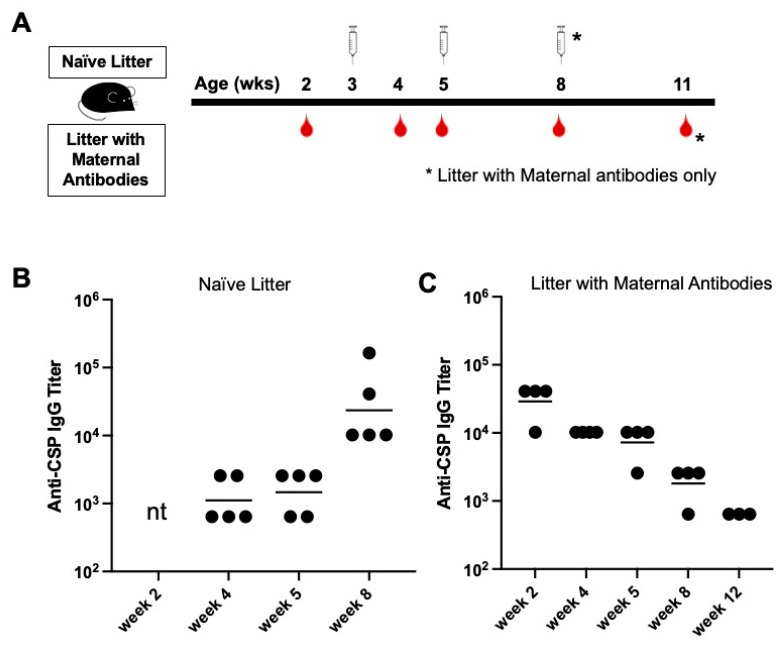
Comparison of antibody responses to CIS43 VLP immunization in mice with and without maternal antibodies. (**A**) Experimental set-up. Mice from a naïve litter and from a litter with maternal antibodies were immunized intramuscularly with 5 µg of CIS43 VLPs at the indicated ages. Sera were collected at the indicated timepoints. (**B**) Anti-CSP IgG endpoint titers in naïve pups (from unimmunized dams) at 4, 5, and 8 weeks of age. (**C**) Anti-CSP IgG endpoint titers in pups from CIS43 VLP-immunized dams at 2, 4, 5, 8, and 12 weeks of age. Each data point represents an individual mouse and lines denote the geometric mean titer of each group. nt, not tested.

To delineate the possible effect of CIS43 VLP-elicited maternal antibodies on de novo immunization, pups of dams immunized with CIS43 VLPs were randomized into different treatment groups. First, we determined whether newborn pups with circulating maternal antibodies would respond to vaccination with CIS43 VLPs. Pups with maternal antibodies were immunized with 5 µg of CIS43 VLPs shortly prior to weaning (at age three weeks) and then were boosted at five weeks of age (Figure 2A). As controls, we also vaccinated a group of age-matched pups born to an unvaccinated dam at the same age. Anti-CSP IgG titers were determined at multiple timepoints up to 12 weeks of age. CIS43 VLPs were immunogenic in naïve young mice (Figure 2B), demonstrating that young mice produce antibody responses upon immunization with CIS43 VLPs. However, there was a complete lack of antibody response in young mice with high levels of circulating maternal antibodies (Figure 2C). These mice received a third immunization at 8 weeks of age, but this booster dose also failed to increase anti-CSP antibody levels. Instead, anti-CSP IgG titers in this group declined steadily over time. Thus, these data show that young mice with pre-existing anti-CSP maternal antibodies do not respond to de novo immunization with the same antigen, demonstrating that maternal antibodies inhibit the ability of an antigen to effectively prime naive B cells. 

### 3.3. Neither Increased Dose nor Changing the VLP Platform Overcome Maternal Antibody-mediated Inhibition

**Figure 3:**
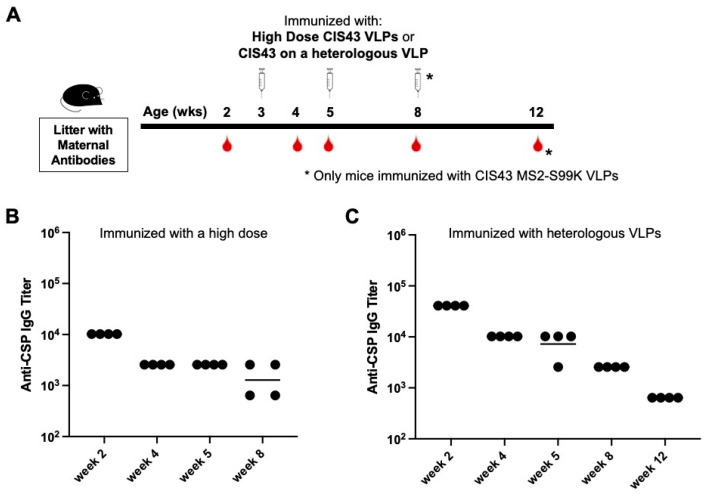
Pups with maternal antibodies do not respond to immunization with high-dose vaccine or CIS43 displayed on a heterologous VLP platform. (**A**) Experimental set-up -- pups from CIS43 VLP-immunized dams were immunized with either (**B**) 20 µg of CIS43 VLPs, or (**C**) 5 µg of CIS43 MS2-S99K VLPs at the indicated ages. In both experiments, sera were collected at the indicated ages and anti-CSP IgG endpoint titers were measured by ELISA. Each data point represents an individual mouse and lines denote the geometric mean titer of each group.

We next asked if maternal antibody-mediated inhibition can be overcome using alternative immunization strategies. First, we hypothesized that increasing the vaccine dose might overcome the level of circulating anti-CSP maternal antibodies, allowing the engagement of B cells and the production of endogenous anti-CSP IgG antibodies. A group of mice was immunized with a four-fold higher dose of CIS43 VLPs (20 µg) at 3 and 5 weeks of age. However, these animals also showed no response to immunization; maternal anti-CSP IgG titers continued to steadily decline over 8 weeks (Figure 3A). Thus, the increased vaccine dose failed to overcome inhibition by maternal antibodies.

In addition to eliciting anti-CSP antibodies, the CIS43 VLP vaccine also elicits antibodies against the Qß VLP platform. To address the possibility that maternal anti-platform antibodies may contribute to the interference with de novo immunization, pups were immunized with a vaccine in which the CIS43 epitope peptide was displayed on an antigenically distinct heterologous VLP. We utilized a modified VLP derived from bacteriophage MS2, called MS2-S99K, which was engineered to display an additional surface-exposed lysine residue on its coat protein subunits. This additional addressable lysine residue enhances the efficiency of the chemical conjugation of the CIS43 peptide. In naïve adult mice, immunization with CIS43 MS2-S99K VLPs elicits similar anti-CSP antibody responses as the Qß-based CIS43 VLPs (Figure S1). Pups with maternal anti-CSP antibodies were immunized with 5 µg of CIS43 MS2-S99K VLPs at 3, 5, and 8 weeks of age. Anti-CSP IgG titers in the group immunized with CIS43 MS2-S99K VLPs also steadily declined over 12 weeks (Figure 3B). Thus, maternal antibody-mediated inhibition is not overcome by using a different VLP platform, indicating that the suppression of de novo antibody responses is likely due to maternal antibodies that target the CIS43 epitope. 

### 3.4. Delaying de novo Immunization Overcomes Vaccine Inhibition Mediated by Anti-CSP Maternal Antibodies

**Figure 4:**
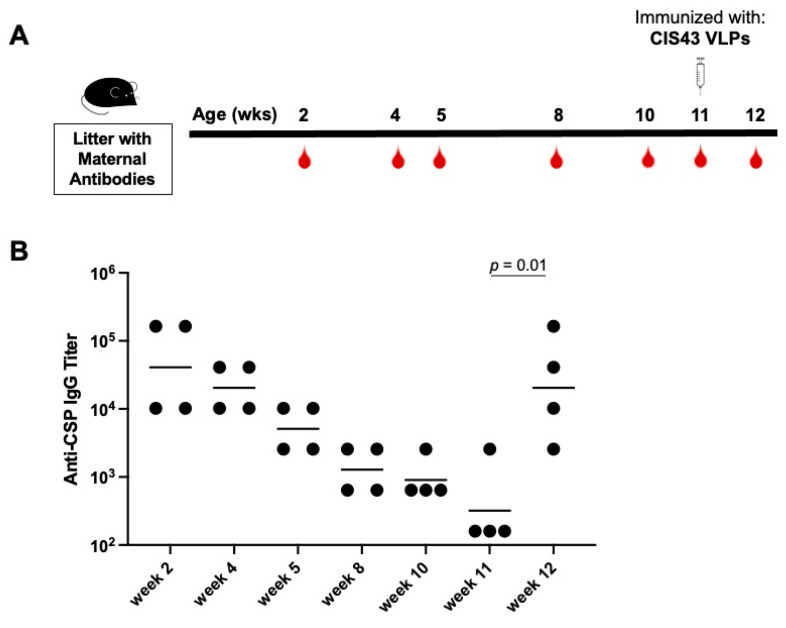
Mice regain responsiveness to CIS43 VLPs as maternal antibody levels wane. (**A**) Experimental set-up. Anti-CSP IgG antibody levels were followed in pups born to CIS43 VLP-immunized dams through 11 weeks of age. At 11 weeks, mice were immunized with 5 µg of CIS43 VLPs. (**B**) Anti-CSP IgG endpoint titers at the indicated ages. Each data point represents an individual mouse and lines denote the geometric mean titer of each group. Data were compared statistically by two-tailed t test using log-transformed values.

We next asked whether delaying immunization of pups until maternal antibody titers waned could restore responsiveness to immunization with CIS43 VLPs. In this experiment, immunization was delayed until mice were >10 weeks old; at this age maternal anti-CSP IgG levels had declined over 40-fold from their peak (at 2 weeks of age; Figure 4). Mice that received delayed immunization with a single 5 µg dose of CIS43 VLPs elicited strong anti-CSP antibody responses; by 12 weeks of age, anti-CSP IgG titers had increased 64-fold (Figure 4). Thus, delaying immunization until pre-existing maternal antibodies decline allows mice to respond to de novo immunization. 

### 3.5. Pre-Existing Antibodies against the VLP Platform Do Not Inhibit Anti-CSP Responses in Adult Mice

**Figure 5:**
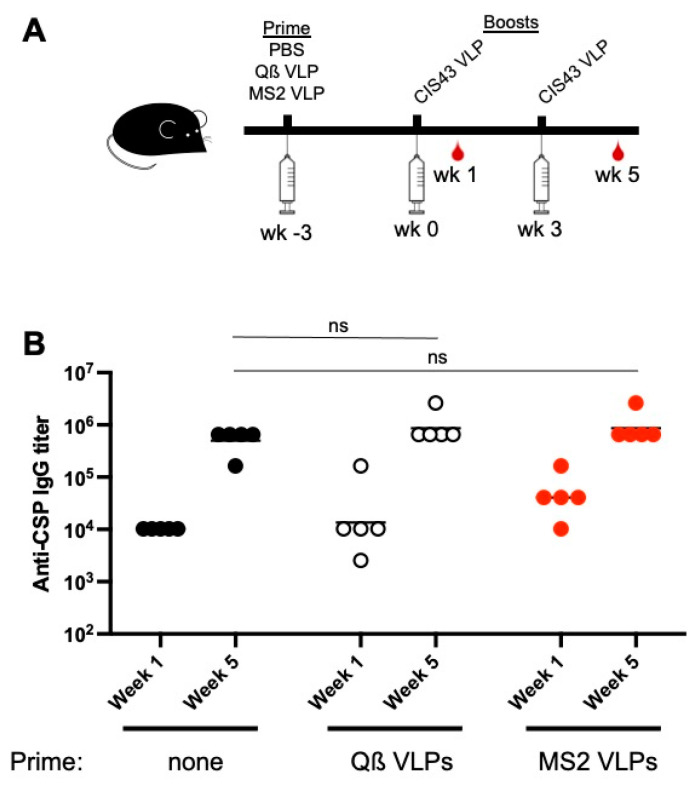
Pre-existing anti-platform immunity does not inhibit vaccine boosting in adult mice. (**A**) Experimental set-up. At week -3, adult mice were primed with PBS, or with 5 µg Qß VLPs or MS2 VLPs. At weeks 0 and 3, mice were immunized with 5 µg CIS43 VLPs. (**B**) Anti-CSP IgG endpoint titers from sera collected at weeks 1 and 5 were measured by ELISA. Each data point represents an individual mouse and lines denote the geometric mean titer of each group. Data were compared statistically by two-tailed t test using log-transformed values. ns, not significant.

Our data demonstrate that maternal-derived antibodies against the CIS43 peptide can inhibit responsiveness to a CIS43 VLP vaccine. Next, we were interested in determining whether pre-existing anti-VLP platform antibodies could inhibit de novo antibody responses against the CIS43 epitope in adult mice. Mice were primed with wild-type Qß VLPs (the CIS43 VLP platform) or with a heterologous VLP platform (MS2 VLPs), and then were boosted twice with CIS43 VLPs. Anti-CSP antibody responses were compared to a group of naïve mice which did not receive a VLP prime. Each of the three groups of mice had similar anti-CSP IgG titers after one and two doses of CIS43 VLPs (Figure 5). Thus, anti-platform antibodies do not inhibit vaccine responsiveness.

## 4. Discussion

Malaria is a disease that causes significant morbidity and mortality in young children. However, infants in malaria-endemic areas are relatively well protected from severe disease through six months of age, possibly due to the presence of maternally derived antibodies against malaria. Although maternal antibodies may provide transient protection from severe disease, they may also potentially interfere with the effectiveness of vaccines against malaria. Here, using a mouse model, we examined whether naturally transferred maternal antibodies inhibit responsiveness to immunization with a VLP-based malaria vaccine that targets the vulnerable CIS43 epitope in the junctional domain of CSP. We show that neonatal mice immunized with CIS43 VLPs generate strong anti-CSP antibody responses, but pups with high levels of maternal antibodies failed to respond to vaccination. This inhibition was not circumvented by administering a higher dose of vaccine, nor by using a heterologous VLP platform. Only when maternal antibody titers were allowed to decline below an inhibitory threshold were mice responsive to vaccination. These data indicate that circulating maternal antibodies can impact the effectiveness of de novo vaccination to a VLP-based immunogen.

Although the suppressive effects of maternal antibodies on the efficacy of live attenuated vaccines, such as the vaccines for rotavirus [36] and measles [37], are well established, fewer studies have examined the effects of maternal antibodies on the immunogenicity of VLP-based vaccines. Nguyen and colleagues showed, in a pig model, that maternal antibodies suppress antibody responses and the generation of memory B cells upon immunization with a rotavirus V2/V6-based VLP vaccine [38]. Similarly, in a large clinical study in China that demonstrated high maternal antibody levels against the Hepatitis B Virus (HBV) impaired antibody responses in infants to the VLP-based HBV vaccine [39], the data were more mixed. However, a different study, performed in pigs, showed that maternal immunization had no effect on the immune responses of neonates to the HBV vaccine [40]. Our data suggest that the level of maternal antibodies is the critical determinant of vaccine efficacy. The use of VLP-based vaccines, which elicit particularly high levels of maternal antibodies, may be particularly prone to suppressing immune response in neonates.

Several different mechanisms have been proposed to explain the suppression of de novo antibody responses by maternal antibodies [11,41]. These include: (1) the elimination of antibody-bound antigen upon Fc-mediated opsonization by phagocytes, (2) epitope masking, whereby antibodies prevent the engagement of antigen by naïve B cells, and (3) feedback inhibition, in which concomitant engagement of the B cell receptor and the inhibitory receptor FcγRIIB inhibits B cell activation. Although determining the mechanism(s) of maternal antibody-mediated suppression was not a primary goal of this study, this could be examined in future studies. For example, it would be interesting to examine whether pups with platform-specific, but not CIS43-specific, maternal antibodies respond to CIS43 VLP immunization. This would allow us to dissect the relative contributions of Fc-mediated inhibition mechanisms versus epitope masking to maternal antibody inhibition. Notably, in adult mice, priming with VLPs did not block responsiveness to CIS43 VLPs, suggesting that Fc receptor-based mechanisms may not be major contributors to antibody-mediated inhibition.

Taken together, these data indicate that the presence of pre-existing anti-CSP maternal antibodies should be considered when determining the precise neonatal timetable of malaria vaccinations to maximize vaccine efficacy. The measles vaccine, for example, is typically administered after 9 months of age to reduce the effects of interference by maternal antibodies [42]. However, delaying immunization runs the risk of leaving infants vulnerable to infection, especially if maternal antibody levels are low. It will be important to investigate other possible approaches for restoring neonatal responsiveness to vaccines in the face of maternal antibodies. These could include the use of novel immunostimulatory adjuvants, emerging vaccine technologies (such as mRNA vaccines), and alternative vaccine delivery strategies (such as approaches that mediate controlled release of vaccines). These future studies will also continue to help clarify the delicate balance that exists between maternal antibodies, their potential to mediate protection from natural infection, and their potential to interfere with immunization.

## Data Availability

All data are presented in this article. Raw ELISA data are available upon request.

## Supplementary Materials

The following supporting information can be downloaded at: https://www.mdpi.com/article/10.3390/biom13020202/s1, Figure S1: CIS43 displayed on Qß VLPs and MS2 S99K VLPs elicit similar antibody responses.
