# Supplementary material for: The Immunogenicity of a VLP-based Malaria Vaccine Targeting CSP in Pregnant and Neonatal Mice
Source: Biomolecules. 2023 Jan 19;13(2):202. doi: 10.3390/biom13020202 (PMC9953288; doi:10.3390/biom13020202)
Supplement: Supplementary file 1 [file biomolecules-13-00202-s001.zip › biomolecules-2172533-supplementary.pdf]

Supplementary Information

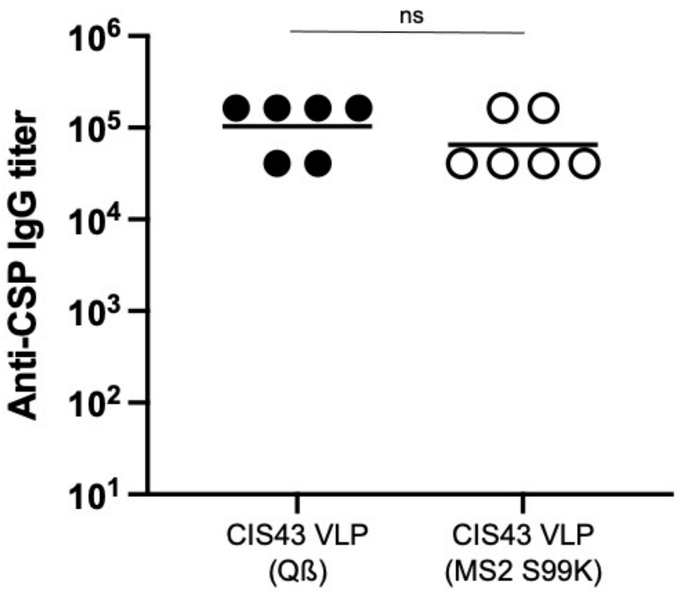

Supplemental Figure S1. CIS43 displayed on Qß VLPs and MS2 S99K VLPs elicit similar antibody responses. Adult mice received three immunizations (at three-week intervals) with 5 µg of CIS43 VLPs or MS2-S99K CIS43 VLPs. Sera were collected two weeks after the final immunization and anti-CSP IgG endpoint dilution titers were measured by ELISA. ns, not significant.
